# Supplementary material for: Patterns of Intron Gain and Loss in Fungi
Source: PLoS Biol. 2004 Nov 30;2(12):e422. doi: 10.1371/journal.pbio.0020422 (PMC532390; doi:10.1371/journal.pbio.0020422)
Supplement: Table S1 — Also available at http://genes.mit.edu/NielsenEtAl/. (4.3 MB ZIP). [file pbio.0020422.st001.zip › NielsenEtAl/html/368.html]

AN9106.1.NCU03862.1.MG00773.1.FG10020.1


```
 CLUSTAL W (1.82) Multiple Sequence Alignments - Introns Inserted


Sequence 1: AN9106.1	394 aa
Sequence 2: FG10020.1	351 aa
Sequence 3: NCU03862.1	392 aa
Sequence 4: MG00773.1	357 aa
Alignment Length: 489 aa
Number Identitical Residues: 99 aa
Alignment Score (without introns) 5149


MG00773.1 	---------------~------------MVVGSALMLRPVPPLVA----------SKPG-
NCU03862.1	MADCRSARSAPAADT0CRDWNAHMPLICLTEPNALKRGTTVPIYCGEVQVEVGMETPPGK
FG10020.1 	---------------~--------------------MSPQHSIVS---------------
AN9106.1  	---------------~-----------------------MIASCG---------------
          	                                         .                  

MG00773.1 	SGFYWQRR~LFNLAGSTNITVSACKP--------------SA~KRFISETP--LRLESSG
NCU03862.1	SDVVLQDY0VTLPASASSHIPSSCSPGRSPLPGPEQSTPRNT1RRYFGASPTCRRQSLDP
FG10020.1 	STHAIQKS~MNRSIIWRKAPVPTLVKYP-----------YRK~QRPIVQCRSFTYSRCVG
AN9106.1  	SASALQSS~C---------AISCCPSFR-----------YSL~LG--RTIRQFS-SRSGR
          	*    *               .                                      

MG00773.1 	QDDPRLKEVLGRTIADDFAKLRSDYA1SPKYPVVLAHGLLGFSTLELAGHLLPPIEYWHG
NCU03862.1	RNNFAPKEYLGKEIVDDYAILRDQYE1TPENPIVLAHGLLGFAELKLAGSFLPSIHYWHG
FG10020.1 	REFDPRIKAVGRAISDDYALIREKYD1TPKYPIVLAHGLFGFAELSLS-PYFPSVEYWHG
AN9106.1  	ASPDPRLEDFGKIIRDEYALVREHYD1TPKHPIILAHGLLGFSELRLAGPLLPGIHYWRG
          	 .     : .*: * *::* :*..*  :*: *::*****:**: * *:.  :* :.**:*

MG00773.1 	VRTALEARGVTVITPAVPPSASIAVRAAKLATQIADSVPSGT~PVNIIA~HSMGGLDARL
NCU03862.1	IKDALSRRNCEVITTTVPPSSSVEQRAAELARGIEARAQ-GK~SVNIIA~HSMGGLDARY
FG10020.1 	IRDALTAQGATVLTPAVPPSSSIADRAAALRSALEAHAPAPE~AVTIVA~HSMGGLDARY
AN9106.1  	IKEAFSLKGVEVIAASVPPSASIEMRAKVLAETIASAARGRE0STDIAC2----GLDARY
          	:: *:  :.  *::.:****:*:  **  *   :   . .   .. * .     ***** 

MG00773.1 	LVSPAFADQLSSVTAERLNVASLVTISTPHRG~STYAEHVINLLGP~---QRVEGARRML
NCU03862.1	MIS-----QLKPASVR---VKSLVTVATPHHG~STFADYLMDEIGP~---ERLPSLYNIW
FG10020.1 	MLS-----HLEPLPVR---VAALVTVATPHRG~SAFADYLLDEEVS~PVHHYLPQLYKTL
AN9106.1  	MIT-----HLKPENFK---VLSLTTIATPHRG1SAVADYLLQQIGD1---DRLAQLYYLL
          	:::     :*..   .   * :*.*::***:* *: *:::::        . :       

MG00773.1 	RTTMGWDNTEAFEQLTPRYMKEVFNPATPDHPDTR~YFSYGAMTDGRPRLFSPFRASHKI
NCU03862.1	ERITGFE-TGAFNQLTKRYMTEDFNPKTPDDPEVR~YFSYGAMVHEKPPLLSPFRLSHGI
FG10020.1 	ERAG--LGTAAFSQLTRRYMAEEFNPSTPDQPDVR~YFSYGAAVER-PALLSPFRHPHAV
AN9106.1  	EKIR--IESGAFSQLTCEYMEKTFNPQTPNIEDIR2YYSYGAVME--PRFWSVFRLSHRL
          	.       : **.*** .** : *** **:  : * *:****  .  * : * ** .* :

MG00773.1 	IQAAEGPNDGLVSVESSKWGTYEGTVVGVSHLALINW2TNRLGWTVRKWLGQEET2YVGE
NCU03862.1	IHGIEGPNDGLVSVQSSRWGTYKGTLSGVNHLDLINW~SNRFRFTLQRWMGRPPT~----
FG10020.1 	MTQAEGPNDGLVSVESSSWGTYKGTLLGVSHLDLINW~SNRVGWTVREWMGIKKN~FNAI
AN9106.1  	LQQLEGYNDGLVSVASSKWGAYKGTLMGVSHLDLINW~SNRLKWLAGEITGQRPN2FNAV
          	:   ** ******* ** **:*:**: **.** **** :**. :   .  *   .   . 

MG00773.1 	KGIVG---~---------------------------------------------------
NCU03862.1	--------~---------------------------------------------------
FG10020.1 	AFYLDIAD~-----------------------MLAKEGF---------------------
AN9106.1  	AFYLDIAE1KPYGVYLGYAHVREEAGQHSDMHTFSDSPFLGCLTVVTIRQQAVSSQVDWT
          	    . :. .. .   . :   ..:.. :.    :..   .. :  :  ..: ::. . :

MG00773.1 	---------------------
NCU03862.1	---------------------
FG10020.1 	---------------------
AN9106.1  	NRGRQGWRCIWIMAMGTPGVA
          	. . ..  .    : .:.. :
```
